# Recognition and management of intra-abdominal hypertension and abdominal compartment syndrome; a survey among Dutch surgeons

**DOI:** 10.1007/s00068-016-0637-x

**Published:** 2016-02-22

**Authors:** Steven G. Strang, Esther M. M. Van Lieshout, Roelof A. Verhoeven, Oscar J. F. Van Waes, Michael H. J. Verhofstad, IAH-ACS Study Group

**Affiliations:** Trauma Research Unit Department of Surgery, Erasmus MC, University Medical Center Rotterdam, P.O. Box 2040, 3000 CA Rotterdam, The Netherlands

**Keywords:** Intra-abdominal pressure, Intra-abdominal hypertension, Abdominal compartment syndrome, Management, Questionnaire

## Abstract

**Purpose:**

Intra-abdominal hypertension (IAH) and Abdominal compartment syndrome (ACS) are relatively rare, but severe complications. Although many advances were made in recent years, the recognition and management remain subject of debate. The aim of this study was to determine the current state of awareness, knowledge and use of evidence-based medicine regarding IAH and ACS among Dutch surgeons.

**Methods:**

A literature-based and expert consensus survey was developed. One surgeon in every hospital in The Netherlands was asked to complete the online questionnaire.

**Results:**

Sixty of 87 (69 %) invited surgeons completed the questionnaire. Intra-abdominal pressure (IAP) was measured using intra-vesical methods by 55 (98 %) respondents. Diuretics (*N* = 38; 63 %) and laparotomy (*N* = 33; 55 %) were considered useful treatments for IAH or prevention of ACS by a majority. Only 16 (27 %) respondents used these guidelines in daily practice, and 37 (62 %) respondents are willing to do so. Although 35 (58 %) surgeons agreed that IAH is only a symptom, not requiring treatment. Forty-one percent of experienced respondents suggested that prevalence of ACS remained unchanged. Nearly all respondents (*N* = 59; 98 %) believed that open abdomen management improves patient outcomes, many (*N* = 46; 77 %) confirm the high complications rate of this treatment.

**Conclusion:**

The definitions of IAH and ACS and the related diagnostic and therapeutic challenges are relatively well known by Dutch surgeons. Despite limited use of the evidence-based guidelines, the willingness to do so is high. Most respondents favor open abdomen treatment for patients with imminent ACS, despite the high complication rates associated with this treatment.

## Introduction

Abdominal compartment syndrome (ACS) is a severe, but relatively rare complication. IAH is more common and can proceed into ACS in some of cases. Over recent years many advances regarding the recognition and management of ACS have been made. Nonetheless, randomized controlled trials on the subject are still scarce. Current management of ACS is based upon the up-to-date, evidence-based recommendations provided by the World Society of the Abdominal Compartment Syndrome (WSACS) [1]. The strength of these recommendations is of varying quality. As a result, the management of ACS is still subject of debate and differs across hospitals.

Multiple studies have been conducted to identify the then current state of awareness, knowledge and use of evidence-based medicine regarding IAH and ACS. One of the most noticeable findings of these studies was that the awareness of IAP measurements and treatment options of IAH and ACS was generally low [2–9]. In addition, cut-off points for treatment of ACS are poorly known or understood [3, 10–13]. There is little agreement on the indications for open abdomen treatment and what type of temporary abdominal closure devices should be used [14–18]. Most recent studies conclude that awareness among health care providers improved over recent years, but guidelines are still not uniformly applied or knowledge was inadequate [19–21].

The most recent survey was performed in 2010. Since then, new developments, such as the introduction of updated WSACS guidelines in 2013, may have improved outcome. Quality of previous questionnaires was variable. The response rates of these questionnaires ranged from 26 to 90 %. Other limitations were duration of more than 2 years and most studies were carried out by a wide variety of health care workers. Only six specifically focused on surgeons, yet surgeons ultimately decide whether or not to apply an open abdomen decompression [2–4, 8, 14, 15]. No comparable surveys have been performed in The Netherlands.

The primary aim of this study was to identify the current state of awareness, knowledge and use of evidence-based medicine regarding IAH and ACS among Dutch surgeons. Secondary aims were to identify the current annual number of ACS cases per hospital and, to assess outcome of ACS patients.

## Methods

This questionnaire study was conducted and reported in accordance with the guidelines for survey research of Bennett et al. [22].

### Ethical statement

The current study used data that were obtained from surgeons using a survey. The questionnaire was anonymous. An independent officer of data and privacy protection in our hospital reviewed the survey procedure and confirmed that participants’ anonymity was protected. Since patients were not involved in the study, the institutional Medical Research Ethics Committee did not have to review the protocol.

### Questionnaire

The questionnaire was based upon a previously published questionnaire by the WSACS study group [21]. Key questions were adopted and response options were added to make them more up-to-date. The questionnaire was drafted in Dutch and pretested by a panel of five experts and critically appraised on relevance, completeness, and style (OJFVW, MHJV, RSB, DHB, and KAK). The final version of the structured questionnaire consisted of five parts with a total of 29 questions; one part for participant’s information and four parts for questions related to (1) IAP measurement, (2) IAH, (3) ACS, (4) open abdomen treatment and abdominal closure techniques. The full questionnaire is available in English (Appendix 1).

### Selection of respondents

Surgical department of all Dutch hospitals with ICU facilities (*N* = 87) was asked to provide the name of the surgeon with the most ICU affinity. If a hospital had multiple locations with ICU facilities, only one surgeon was selected. All named surgeons were approached by telephone and informed about the purpose and method of the survey. Since one surgeon in every hospital throughout the country was selected, the targeted group of surgeons was presumed a representative cross-section of the care which patients in The Netherlands receive. Dutch surgical departments are relatively well informed and the rate of evidence-based guideline implementation is high. The results of this survey are therefore applicable for to Western European standards. For this survey, a sample size calculation was considered unnecessary.

### Distribution of survey

The questionnaire was distributed online using LimeSurvey software [Version 2.05+, LimeSurvey Project Team, Carsten Schmitz (2015), LimeSurvey Project Hamburg, Germany]. After obtaining verbal informed consent, a link to the questionnaire with unique and secure access codes was sent by email. This first invitation was sent on January 29, 2015. Reminders were sent every 2 weeks until the survey was closed on April 13, 2015. An opt-out link was clearly marked, the questionnaire could also be sent by mail or email if requested.

### Data

Data were stored online by a secured function of the software used. Following survey closure, data were downloaded to an SPSS file. Questionnaires that were completed on paper were entered manually into the SPSS database. Only complete data sets were included in the analysis.

### Analysis

All data were of categorical nature and are shown as numbers with corresponding percentages. Descriptive analysis was performed in SPSS version 21.0 (SPSS Statistics for Windows, Released 2012, Armonk, New York, IBM Corporation). No comparisons were made with previously performed surveys since differences between questionnaires and populations were considered too large.

## Results

### Respondents

**Fig. 1:**
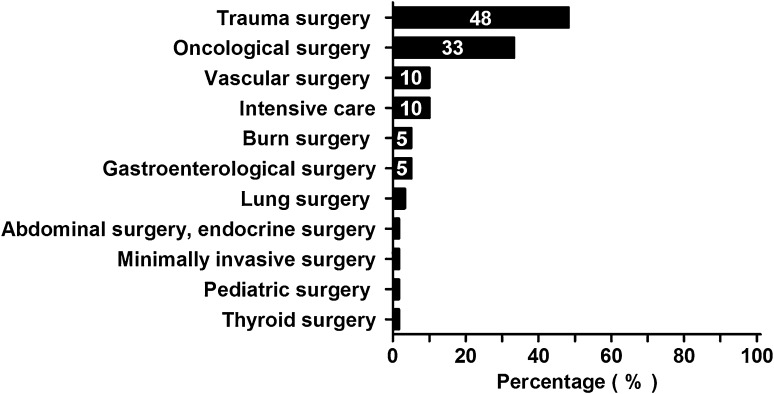
Primary focus of respondents. Primary focus of respondents is arranged on the *y*-axis from highest to lowest frequency. Percentages of all respondents are shown in the *bars*

Sixty surgeons completed the questionnaire (response rate: 69 %). Ten partial responses were excluded. Most respondents had a primary focus on trauma surgery (*N* = 29; 48 %) or oncological surgery (*N* = 20; 33 %, Fig. 1). The majority (*N* = 38; 63 %) had over 10 years of surgical experience and more than half of respondents worked in a general teaching hospital (*N* = 34; 57 %).

### Intra-abdominal pressure measurements

IAP measurements were performed in 58 (96 %) of the hospitals. Forty-seven (78 %) respondents claimed to know the difference between IAH and ACS, and 57 (95 %) respondents had seen at least one patient with ACS.

**Fig. 2:**
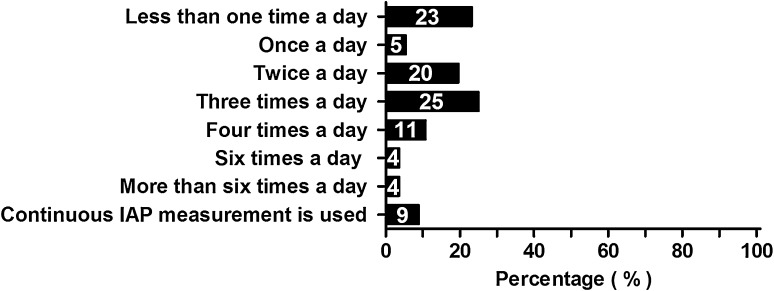
Number of IAP measurements performed daily in the individual patient. Percentages of all respondents are shown in the *bars*

Fifty-five (98 %) respondents use intra-vesical methods for IAP measurement. The largest group of respondents (*N* = 14; 25 %) measures IAP three times daily on average (Fig. 2).

**Fig. 3:**
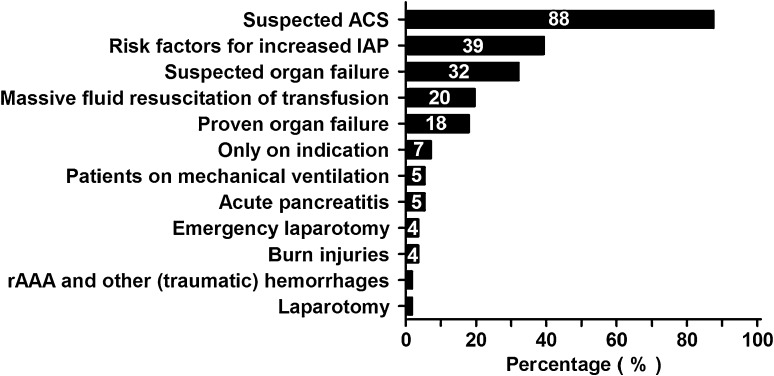
Percentage of patients in which IAP measurements are performed more or less routinely (patients with or after a/an:). Percentages of all respondents are shown in the *bars*

Forty-nine (88 %) respondents wait with measuring of IAP until there is a clear suspicion for ACS, and 22 (39 %) respondents start measurements as soon as risk factor(s) for ACS are identified (Fig. 3).

### Intra-abdominal hypertension

**Fig. 4:**
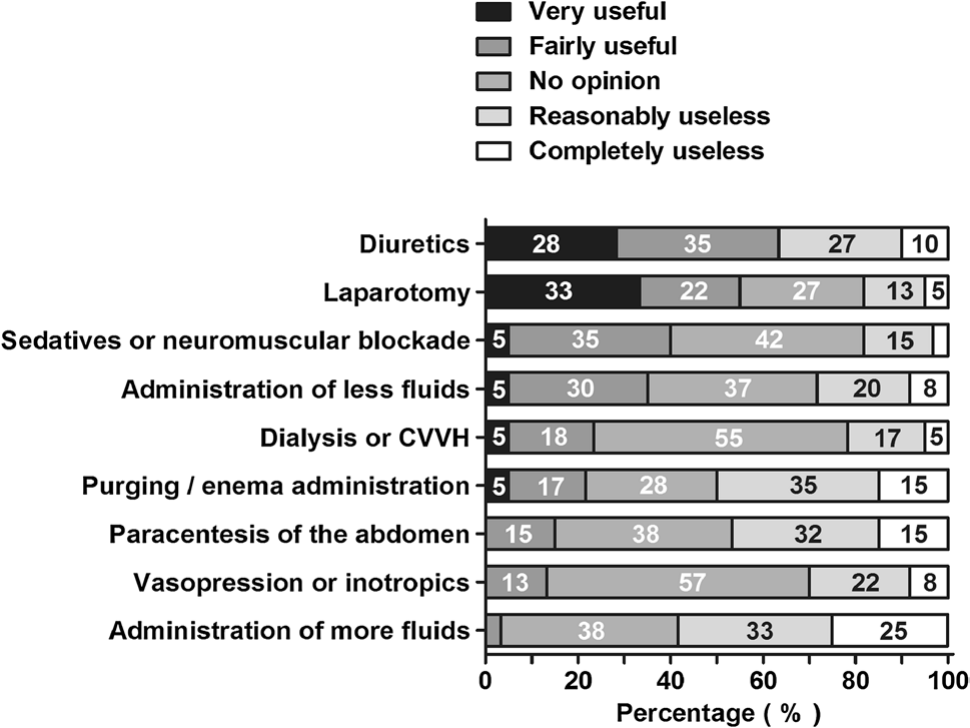
Usefulness of treatments for IAH to prevent ACS. Therapy options are arranged from highest to lowest summed percentage of very useful and fairly useful. Percentages of all respondents are shown in the *bars*

Forty-two (70 %) respondents claimed to use the definition of IAH as set by the WSACS (Table 1). Of the seven treatment options listed for IAH, only diuretics (*N* = 38; 63 %) and laparotomy (*N* = 33; 55 %) were considered very useful or fairly useful by the majority of respondents (Fig. 4). Thirty-five (58 %) respondents agreed to the statement that IAH is only a symptom and as such needs no treatment.

**Table 1:** Used definition for IAH (not ACS)

	*N*	*%*
An IAP of ≥12 mmHg, as stated by the WSACS	42	70
An IAP of >18 mmHg	1	2
An IAP of >20 mmHg	1	2
Ongoing or increasing IAP at multiple measurements	1	2
No definition	15	25

### Abdominal compartment syndrome

**Fig. 5:**
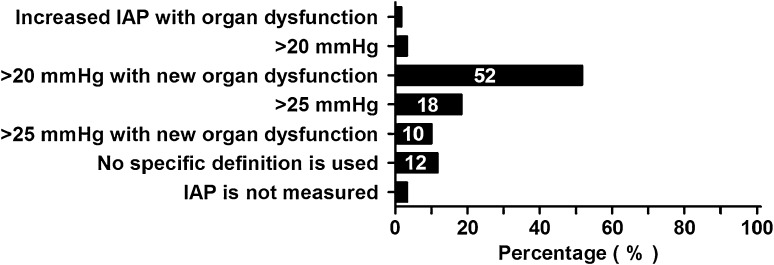
Definition used for ACS (not IAH, an IAP of:). Percentages of all respondents are shown in the *bars*

For ACS, the majority of respondents (*N* = 31, 52 %) used the definition as proposed by the WSACS (Fig. 5). It was noteworthy that 17 (28 %) respondents used a higher threshold for ACS.

Most respondents (*N* = 33; 55 %) were not familiar with the WSACS guidelines for the treatment of ACS. Whereas 27 (45 %) respondents were familiar with the guidelines, only 16 (27 %) actually use them in daily practice. Another 37 (62 %) respondents is willing to do so in the future (Table 2). A minority (*N* = 6; 10 %) disputes that the guidelines improve outcome of patients with ACS.

**Table 2:** Use of WSACS guidelines and recommendations for treatment of abdominal compartment syndrome

	*N*	*%*
This guideline is used	16	27
This guideline is not used, but respondent is willing to do so	37	62
This guideline is not used because it presumably does not improve the outcome of patients	6	10
There is no need for such a guideline	1	2

**Fig. 6:**
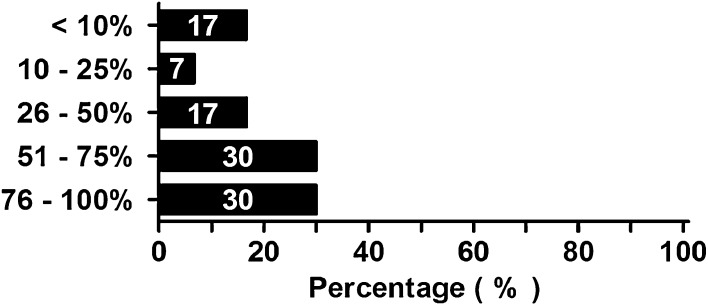
Number of ACS patients per hospital, treated with a surgical abdominal decompression. Percentages of all respondents are shown in the *bars*

Eighteen (30 %) respondents answered that patients with ACS should be treated with surgical decompression in 76 % to 100 % of cases in their hospital (Fig. 6). Another 18 (30 %) indicated that this was done in 51–75 % of cases.

**Fig. 7:**
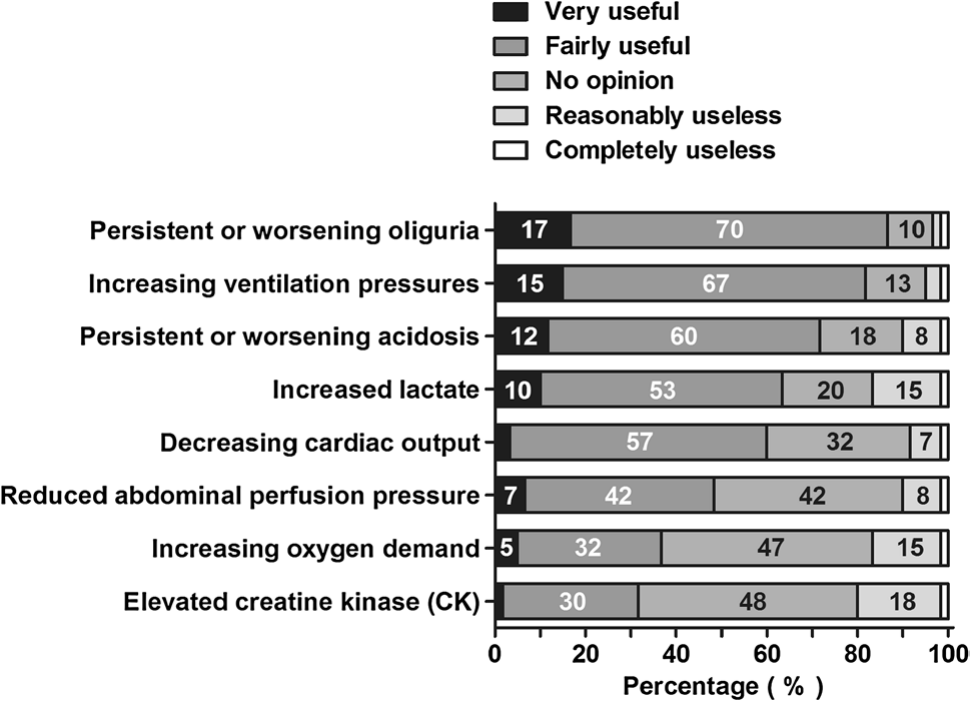
Usefulness of factors in deciding for surgical abdominal decompression (in addition to intra-abdominal pressure). Factors are arranged from highest to lowest summed percentage of very useful and fairly useful. Percentages of all respondents are shown in the *bars*

The vast majority of respondents considered oliguria, ventilation pressure, acidosis, lactate, and cardiac output as relevant when deciding for a surgical abdominal decompression (Fig. 7). A large group (*N* = 26; 43 %) stated that a superior indicator for surgical decompression would be a useful addition into clinical practice (Table 3).

**Table 3:** Need for superior indicators of abdominal decompression (for example a serum marker of hypo-perfusion of abdominal organs)

	*N*	*%*
Yes, there is a need for superior indicators	26	43
I do not know/no opinion	26	43
No, there is no need for superior indicators	8	13

**Fig. 8:**
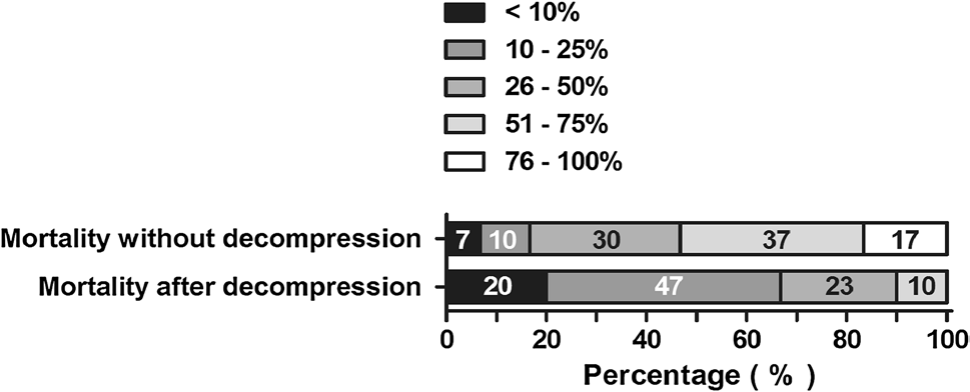
Estimated mortality rate among patients with ACS. Percentages of all respondents are shown in the *bars*

The mortality rate of patients with ACS who are not treated with surgical decompression was estimated between 26 and 50 % by 18 (30 %) respondents and between 51 and 75 % by 22 (37 %) respondents (Fig. 8). If patients with ACS were treated with surgical decompression, the largest group of respondents (*N* = 28; 47 %) estimated a mortality rate of 10–25 %.

### Open abdomen treatment and abdominal closure techniques

Fifty-three (88 %) respondents considered surgical abdominal decompression useful in the prevention of ACS (Table 4). However, the majority felt that ACS may not always be prevented.

**Table 4:** Open abdominal treatment prevents ACS

	*N*	*%*
Yes, always	16	27
Yes, but not always	37	62
I am not sure	6	10
No, never	1	2

**Fig. 9:**
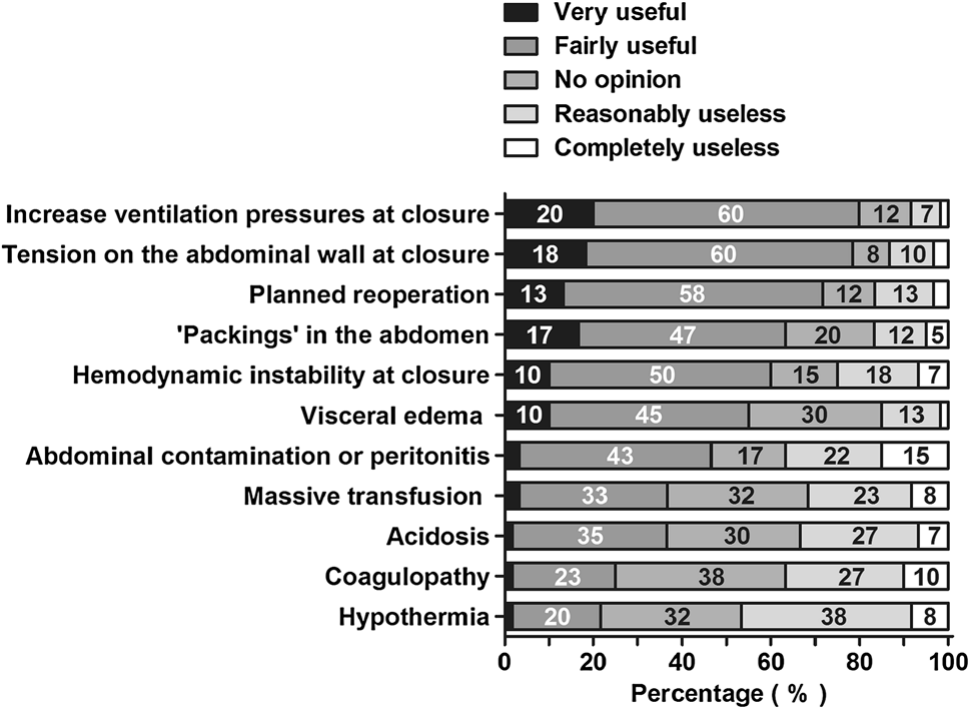
Usefulness of factors in deciding not to close the abdomen after surgical decompression. Factors are arranged from highest to lowest summed percentage of very useful and fairly useful. Percentages of all respondents are shown in the *bars*

The respondents were asked which factors would affect their decision whether or not to close the abdomen after surgical decompression. Most respondents answered that an increase in ventilation pressures is either useful (*N* = 36; 60 %) or very useful (*N* = 12; 20 %) in this decision (Fig. 9). In addition, tension on the abdominal wall while closing the abdomen, planned reoperation, application of abdominal packings, hemodynamic instability at closure and visceral edema were also considered useful by the majority of the respondents.

If primary closure is not possible, several devices are available for temporary closure. Among the respondents, application of a Vicryl^®^ mesh was the most popular method for temporary closure, chosen by 38 (63 %) respondents (Table 5). Many respondents selected multiple methods of temporary closure, 22 (37 %) respondents selected two methods and 18 (30 %) even selected three.

**Table 5:** Used temporary abdominal closure method or devices

	*N*	*%*
Mesh placement (Vicryl^®^)	38	63
Bogota/silo bag	28	47
Abdominal VAC	14	23
Vacuum pack	14	23
Only closure of the fascia	3	5
Closure of the skin (with surgical clamps)	3	5
Closing of the skin with thick suture	1	2
Regular gauze cover	1	2

Percentages add up to more than 100 % because respondents could select more than one answer

The largest group (*N* = 27; 45 %) of respondents prefers definitive abdominal closure in multiple stages (Table 6). An almost equally large group (*N* = 26; 43 %) prefers the component separation technique.

**Table 6:** Used definitive abdominal closure method

	*N*	*%*
Staged closure of the abdomen	27	45
Component separation technique	26	43
Absorbable mesh	22	37
Complete closure of fascia and skin	21	35
Only closure of the fascia	20	33
Non-absorbable mesh	17	28
ABRA system	12	20
Only closure of the skin	5	8
Delayed hernia	1	2
Dual mesh	1	2
Try to prevent non-resorbable materials	1	2

Percentages add up to more than 100 % because respondents could select more than one answer

Almost all respondents (*N* = 59; 98 %) believed that open abdomen management improves patient outcomes, of which the majority (*N* = 46) also acknowledged the high complication rate associated with open abdomen management (Table 7).

**Table 7:** Reply to statement: “Open abdomen treatment improves the outcome of patients with ACS”

	*N*	*%*
Agree	13	22
Agree, but open abdomen treatment is associated with many complications	46	77
Disagree, the complications outweigh the benefits of open abdomen treatment	1	2

## Discussion

This study is the first survey detailing awareness, knowledge, and use of evidence-based medicine and outcome regarding intra-abdominal hypertension and abdominal compartment syndrome among Dutch surgeons. The definitions of the WSACS are well known now, but the clinical practice guidelines of this society are still waiting to be implemented in hospitals. Much disagreement exists today with respect to treatment and outcome of intra-abdominal hypertension and abdominal compartment syndrome among Dutch surgeons.

Ninety-five percent of respondents had previously treated a patient with ACS in their hospital. This was in line with the 97 % reported by Tiwari et al. [12]. IAP measurements were regularly performed in 96 % of the participating hospitals, which was markedly higher than the 31–47 % reported in other surveys [4, 5]. The frequency of IAP measurements, however, varied greatly among hospitals. In 13 (23 %) hospitals, IAP was measured less than once per 24 h. This frequency is rather low since IAP related morbidity can potentially develop or progress within a few hours [23].

There is still no consensus on the management of IAH and ACS. Although many respondents believed that IAH is only a symptom which does not necessarily needs to be treated, several different treatment options for IAH to prevent ACS were considered useful. For example, the use of diuretics and laparotomy are considered valuable. The majority of respondents were indifferent about other treatment options or regarded them as useless. This indifference about IAH treatment has previously been noted by Kimball et al. [2].

Most respondents (88 %) think that surgical decompression could prevent ACS and improve patient outcomes. This is markedly higher than the 60 % of respondents who would recommend decompression laparotomy as reported by Zhou et al. [9]. Despite several indicators for surgical abdominal decompression were believed to be useful, 43 % of our respondents felt the need for a superior indicator.

There is disagreement between respondents and literature regarding temporary abdominal closure (TAC) devices. Respondents reported to prefer mesh assisted TAC. Although evidence is not conclusive, literature slightly favors vacuum assisted techniques [24]. Definitive closure techniques ideally bring the edges of the abdominal fascia together primarily (primary closure). If this is not feasible, simple coverage or functional closure can be provided. These techniques are generally regarded as inferior with respect to patient outcome. Respondents seem to be aware of this, since they mostly preferred staged abdominal closure, followed by the component separation closure technique.

The vast majority of respondents were convinced of the necessity of open abdomen treatment for patients with imminent ACS, even though they were aware of the high complication rate associated with this treatment. They estimate that benefits of open abdomen treatment outweigh the chance of complications. This statement is confirmed by the presumed mortality reduction as result of open abdomen decompression as demonstrated in Fig. 9. The current study confirms there is a large support for this treatment, even though there is disagreement recent literature regarding the benefits of open abdominal decompression in pancreatitis patients with ACS [25, 26].

The strength of the current study is its robust methodology. The survey was based on previous questionnaires, was developed by an expert group, and was repeatedly pretested. Surgeons were kindly, but persistently urged to participate. The online software enables swift responding and easy data collection. The nationwide coverage of this survey is also considered a strength.

Taken into account the 10 incomplete responses, the response rate of 69 % was fairly high. This number is at the upper end of response rates of the previously performed surveys on IAH and ACS (range 26–90 %) [2–21].

The skewed distribution of the primary focus of respondents can be regarded a limitation of this study, but is representative of the clinical practice in The Netherlands. The overrepresentation of trauma surgeons and oncological surgeons may be the result of the connection between these sub-specializations and intensive care medicine. It was, however, not the intention to approximate a cross-section of all Dutch surgeons, but rather of the care patients actually receive. Assuming that patients will usually be treated by a physician with the most relevant knowledge and experience, we are convinced that the results of this study really demonstrate the awareness and knowledge of the surgeon with the largest relevant experience and knowledge. Another shortcoming is that the estimation of change in ACS occurrence over the last 10 years could be subject to recall bias. Although this was an important question, its outcomes were likely to be inaccurate. For example, six respondents indicated that a decrease in ACS incidence did not occur, while they ticked a lower number of cases category for last year compare than for 10 years ago (Table 2). However, this question does give insights in the perception of the experienced surgeon.

The overall knowledge and implementation of WSACS recommendations were lower than expected. This may be due to the fact that the vast majority of the respondents received their surgical training before the WSACS guidelines were developed. ACS treatment is currently implemented in these training programs. The results of the current study and the implementation in surgical training programs should result in increased awareness in the future.

In conclusion, the definitions of IAH and ACS and related diagnostic and therapeutic challenges are relatively well known among Dutch surgeons. Although use of the WSACS guidelines is currently limited, the willingness to do so is large. The vast majority of respondents are convinced of the necessity of open abdomen treatment for patients with imminent ACS, even though this treatment is associated with high complication rates. To decrease the complication rate, many respondents support the need for a superior indicator for surgical abdominal decompression.

## References

[1] Kirkpatrick AW, Roberts DJ, De Waele J, Jaeschke R, Malbrain ML, De Keulenaer B, Duchesne J, Bjorck M, Leppaniemi A, Ejike JC (2013). Intra-abdominal hypertension and the abdominal compartment syndrome: updated consensus definitions and clinical practice guidelines from the World Society of the Abdominal Compartment Syndrome. Intensive Care Med.

[2] Kimball EJ, Rollins MD, Mone MC, Hansen HJ, Baraghoshi GK, Johnston C, Day ES, Jackson PR, Payne M, Barton RG (2006). Survey of intensive care physicians on the recognition and management of intra-abdominal hypertension and abdominal compartment syndrome. Crit Care Med.

[3] Kirkpatrick AW, Laupland KB, Karmali S, Bergeron E, Stewart TC, Findlay C, Parry N, Khetarpal S, Evans D (2006). Spill your guts! Perceptions of Trauma association of Canada member surgeons regarding the open abdomen and the abdominal compartment syndrome. J Trauma.

[4] De Laet IE, Hoste EA, De Waele JJ (2007). Survey on the perception and management of the abdominal compartment syndrome among Belgian surgeons. Acta Chir Belg.

[5] Burke BA, Latenser BA (2008). Defining intra-abdominal hypertension and abdominal compartment syndrome in acute thermal injury: a multicenter survey. J Burn Care Res..

[6] Otto J, Kaemmer D, Hoer J, Jansen M, Schumpelick V, Strik M, Kuhlen R, Schachtrupp A (2009). Bedeutung des abdominellen Kompartmentsyndroms in Deutschland: Eine Umfrage. Anaesthesist..

[7] Ejike JC, Newcombe J, Baerg J, Bahjri K, Mathur M (2010). Understanding of abdominal compartment syndrome among pediatric healthcare providers. Crit Care Res Pract..

[8] Costa S, Gomes A, Graca S, Ferreira A, Fernandes G, Esteves J, Costa A, Fernandes P, Casteloes P, Maciel J (2011). Sindrome de compartimento abdominal: questionario sobre a sensibilidade dos cirurgioes gerais Portugueses. Acta Med Port.

[9] Zhou JC, Zhao HC, Pan KH, Xu QP (2011). Current recognition and management of intra-abdominal hypertension and abdominal compartment syndrome among tertiary Chinese intensive care physicians. J Zhejiang Univ Sci B..

[10] Nagappan R, Ernest D, Whitfield A (2005). Recognition and management of intra-abdominal hypertension and abdominal compartment syndrome. Crit Care Resusc.

[11] Ravishankar N, Hunter J (2005). Measurement of intra-abdominal pressure in intensive care units in the United Kingdom: a national postal questionnaire study. Br J Anaesth.

[12] Tiwari A, Myint F, Hamilton G (2006). Recognition and management of abdominal compartment syndrome in the United Kingdom. Intensive Care Med.

[13] Biancofiore G, Bindi ML (2008). Measurement and knowledge of intra-abdominal pressure in Italian Intensive Care Units. Minerva Anestesiol.

[14] Mayberry JC, Goldman RK, Mullins RJ, Brand DM, Crass RA, Trunkey DD (1999). Surveyed opinion of American trauma surgeons on the prevention of the abdominal compartment syndrome. J Trauma.

[15] Karmali S, Evans D, Laupland KB, Findlay C, Ball CG, Bergeron E, Stewart TC, Parry N, Khetarpal S, Kirkpatrick AW (2006). To close or not to close, that is one of the questions? Perceptions of Trauma Association of Canada surgical members on the management of the open abdomen. J Trauma.

[16] Choi JY, Burton P, Walker S, Ghane-Asle S (2008). Abdominal compartment syndrome after ruptured abdominal aortic aneurysm. ANZ J Surg..

[17] MacLean AA, O’Keeffe T, Augenstein J (2008). Management strategies for the open abdomen: survey of the American Association for the Surgery of Trauma membership. Acta Chir Belg.

[18] Herrle F, Hasenberg T, Fini B, Jonescheit J, Shang E, Kienle P, Post S, Niedergethmann M (2011). Offenes abdomen 2009. Umfrage zu Behandlungsstrategien des offenen abdomens in Deutschland. Chirurg..

[19] Kaussen T, Steinau G, Srinivasan PK, Otto J, Sasse M, Staudt F, Schachtrupp A (2012). Recognition and management of abdominal compartment syndrome among German pediatric intensivists: results of a national survey. Ann Intensive Care..

[20] Newcombe J, Mathur M, Bahjri K, Ejike JC (2012). Pediatric critical care nurses’ experience with abdominal compartment syndrome. Ann Intensive Care..

[21] Wise R, Roberts DJ, Vandervelden S, Debergh D, De Waele JJ, De Laet I, Kirkpatrick AW, De Keulenaer BL, Malbrain ML. Awareness and knowledge of intra-abdominal hypertension and abdominal compartment syndrome: results of an international survey. Anaesthesiol Intensive Ther. 2014. [Epub ahead of print] PubMed PMID: 25251947. 10.5603/AIT.2014.0051 25251947

[22] Bennett C, Khangura S, Brehaut JC, Graham ID, Moher D, Potter BK, Grimshaw JM (2010). Reporting guidelines for survey research: an analysis of published guidance and reporting practices. PLoS Med..

[23] Rodas EB, Malhotra AK, Chhitwal R, Aboutanos MB, Duane TM, Ivatury RR (2005). Hyperacute abdominal compartment syndrome: an unrecognized complication of massive intraoperative resuscitation for extra-abdominal injuries. Am Surg..

[24] van Boele Hensbroek P, Wind J, Dijkgraaf MG, Busch OR, Goslings JC (2009). Temporary closure of the open abdomen: a systematic review on delayed primary fascial closure in patients with an open abdomen. World J Surg.

[25] Ke L, Ni HB, Tong ZH, Li WQ, Li N, Li JS (2013). The importance of timing of decompression in severe acute pancreatitis combined with abdominal compartment syndrome. J Trauma Acute Care Surg..

[26] Mentula P, Leppaniemi A (2014). Position paper: timely interventions in severe acute pancreatitis are crucial for survival. World J Emerg Surg.

